# Supplementary material for: Deciphering multi-way interactions in the human genome
Source: Nat Commun. 2022 Sep 20;13:5498. doi: 10.1038/s41467-022-32980-z (PMC9489732; doi:10.1038/s41467-022-32980-z)
Supplement: Supplementary file 1 — Supplementary Information [file 41467_2022_32980_MOESM1_ESM.pdf]

Deciphering Multi-way Interactions in the Human Genome

Gabrielle A. Dotson[1,†], Can Chen[2,3,4,†], Stephen Lindsly[1,†], Anthony Cicalo[1], Sam Dilworth[5], Charles Ryan[7,8], Sivakumar Jeyarajan[1], Walter Meixner[1], Cooper Stansbury[1], Joshua Pickard[1], Nicholas Beckloff[6], Amit Surana[9], Max Wicha[10], Lindsey A. Muir[1], and Indika Rajapakse[1,2,*]

[1]Department of Computational Medicine and Bioinformatics, University of Michigan, Ann Arbor, MI 48109 USA
[2]Department of Mathematics, University of Michigan, Ann Arbor, MI 48109 USA
[3]Department of Electrical Engineering and Computer Science, University of Michigan, Ann Arbor, MI 48109 USA
[4]Channing Division of Network Medicine, Brigham and Women's Hospital and Harvard Medical School, Boston, MA 02115 USA
[5]iReprogram, Ann Arbor, MI 48105 USA
[6]Oxford Nanopore Technologies, Oxford, OX4 4DQ UK
[7]Medical Scientist Training Program, University of Michigan, Ann Arbor, MI 48109 USA
[8]Program in Cellular and Molecular Biology, University of Michigan, Ann Arbor, MI 48109 USA
[9]Raytheon Technologies Research Center, East Hartford, CT 06108 USA
[10]Department of Hematology/Oncology, University of Michigan, Ann Arbor, MI 48109 USA
[†]These authors contributed equally to this work.
[*]To whom correspondence should be addressed (indikar@umich.edu).

1 Supplementary Methods

Supplementary Algorithm 1: Hypergraph incidence matrix construction

Input: Aligned Pore-C data
for each multi-way contact j **do**
  if multi-way contact contains locus i **then**
    $\mathbf{H}(i, j) = 1$
  else
    $\mathbf{H}(i, j) = 0$
  end if
end for
Return: Hypergraph incidence matrix $\mathbf{H} \in \mathbb{R}^{n \times m}$ where n is the total number of loci, and m is the total number of multi-way contacts.

Supplementary Algorithm 2: Identification of Transcription Clusters

Input: Hypergraph incidence matrix $\mathbf{H}$, gene expression $\mathbf{R}$ (RNA-seq), RNA Pol II $\mathbf{P}$ (ChIP-seq), chromatin accessibility $\mathbf{C}$ (ATAC-seq), transcription factor binding motifs $\mathbf{B}$
for each multi-way contact j in $\mathbf{H}$ **do**
  if all loci are accessible from $\mathbf{C}$ and ≥ 1 locus has Pol II binding from $\mathbf{P}$ **then**
    multi-way contact j from $\mathbf{H}$ is added to the set of potential transcription clusters $\mathbf{T}_p$
  end if
end for
for each potential transcription cluster k in $\mathbf{T}_p$ **do**
  if loci contain ≥ 2 expressed genes from $\mathbf{R}$ and the majority of these genes have ≥ 1 common TFs from $\mathbf{B}$ **then**
    multi-way contact k from $\mathbf{T}_p$ is added to the set of transcription clusters $\mathbf{T}_c$
  end if
  if loci contain ≥ 2 expressed genes from $\mathbf{R}$ and the majority of these genes have ≥ 1 common MRs from $\mathbf{B}$ **then**
    multi-way contact k from $\mathbf{T}_p$ is added to the set of transcription clusters $\mathbf{T}_s$
  end if
end for
Return: Potential transcription clusters $\mathbf{T}_p$, transcription clusters $\mathbf{T}_c$, and specialized transcription clusters $\mathbf{T}_s$.

Supplementary Algorithm 3: Hypergraph Entropy

Input: Hypergraph incidence matrix $\mathbf{H} \in \mathbb{R}^{n\times m}$

Construct the hypergraph Laplacian matrix $\mathbf{L} = \mathbf{D} - \mathbf{H}\mathbf{E}^{-1}\mathbf{H}^\top \in \mathbb{R}^{n\times n}$ where $\mathbf{D} \in \mathbb{R}^{n\times n}$ is a diagonal matrix containing the degrees of nodes along its diagonal, and $\mathbf{E} \in \mathbb{R}^{m\times m}$ is a diagonal matrix containing the orders of hyperedges along its diagonal

Compute the eigenvalues λ_i of $\mathbf{L}$ using eigendecomposition

Normalize the eigenvalues $\bar{\lambda}_j = \frac{\lambda_j}{\sum_{i=1}^n \lambda_i}$

Compute the hypergraph entropy

$$S = -\sum_j \bar{\lambda}_j \ln \bar{\lambda}_j$$

Return: Hypergraph entropy S.

Supplementary Algorithm 4: Comparing Hypergraphs

Input: Two hypergraph G_1 and G_2 with incidence matrices $\mathbf{H}_1 \in \mathbb{R}^{n\times m_1}$ and $\mathbf{H}_2 \in \mathbb{R}^{n\times m_2}$

Construct the normalized hypergraph Laplacian matrices

$$\tilde{\mathbf{L}}_i = \mathbf{I} - \mathbf{D}_i^{-\frac{1}{2}}\mathbf{H}_i\mathbf{E}_i^{-1}\mathbf{H}_i^\top\mathbf{D}_i^{-\frac{1}{2}} \in \mathbb{R}^{n\times n},$$

where $\mathbf{I} \in \mathbb{R}^{n\times n}$ is the identity matrix, $\mathbf{E}_i \in \mathbb{R}^{m_i\times m_i}$ is a diagonal matrix containing the orders of hyperedges along its diagonal, and $\mathbf{D}_i \in \mathbb{R}^{n\times n}$ is a diagonal matrix containing the degrees of nodes along its diagonal, for $i = 1, 2$

Compute the hypergraph distance

$$d = \frac{1}{n}\left(\sum_{i=1}^n |\lambda_{1j} - \lambda_{2j}|^p\right)^{\frac{1}{p}},$$

where λ_{ij} is the jth eigenvalue of $\tilde{\mathbf{L}}_i$ for $i = 1, 2$, and $p \geq 1$

Other options: Hamming distance and DeltaCon distance (Methods)

Return: Hypergraph distance d between G_1 and G_2.

Supplementary Algorithm 5: Permutation Test for Hypergraph Distance

Input: Two hypergraph G_1 and G_2, a prescribed significant level α

Null hypothesis H_0: G_1 and G_2 are similar, and alternative hypothesis H_1: G_1 and G_2 are dissimilar

Randomly generate N number of hypergraph $\{\mathsf{R}_i\}_{i=1}^N$ that are similar to G_1 ("similar" means similar number of node degree and hyperedge size distribution)

Construct the the background distribution by measuring the hypergraph distances between G_1 and R_i for $i = 1, 2, \ldots, N$

Compute the actual hypergraph distance between G_1 and G_2

Obtain the p-value by calculating the proportion of distances that are greater than the actual distance in the background distribution

if p-value $\leq \alpha$ **then**

    Reject the null hypothesis H_0

end if

Return: Permutation test result of whether G_1 and G_2 are similar or dissimilar.

Supplementary Algorithm 6: Identification of Transcription Clusters with Enhancers

Input: Hypergraph incidence matrix $\mathbf{H}$, gene expression $\mathbf{R}$ (RNA-seq), RNA Pol II $\mathbf{P}$ (ChIP-seq), chromatin accessibility $\mathbf{C}$ (ATAC-seq), enhancer locations $\mathbf{E}$, transcription factor binding motifs $\mathbf{B}$
for each multi-way contact j in $\mathbf{H}$ **do**
  if all loci are accessible from $\mathbf{C}$ and ≥ 1 locus has Pol II binding from $\mathbf{P}$ **then**
    multi-way contact j from $\mathbf{H}$ is added to the set of potential transcription clusters $\mathbf{T}_p$
  end if
end for
for each potential transcription cluster k in $\mathbf{T}_p$ **do**
  if loci contain ≥ 2 expressed genes from $\mathbf{R}$ which have ≥ 1 common TFs from $\mathbf{B}$ and ≥ 1 enhancer from $\mathbf{E}$ **then**
    multi-way contact k from $\mathbf{T}_p$ is added to the set of transcription clusters $\mathbf{T}_c$
  end if
  if loci contain ≥ 2 expressed genes from $\mathbf{R}$ which have ≥ 1 common MRs from $\mathbf{B}$ and ≥ 1 enhancer from $\mathbf{E}$ **then**
    multi-way contact k from $\mathbf{T}_p$ is added to the set of transcription clusters $\mathbf{T}_s$
  end if
end for
Return: Potential transcription clusters $\mathbf{T}_p$, transcription clusters $\mathbf{T}_c$, and specialized transcription clusters $\mathbf{T}_s$

2 Supplementary Notes

Supplementary Note 1: Hypergraph Distance Measures (HDMs). The Hamming distance compares the neighborhood of each node, i.e. direct neighbours, and thus is a local similarity measure. The spectral distance measures differences in the graph structure at a global scale as it is based on eigenvalues of the normalized Laplacian $\tilde{\mathbf{L}}$ which captures global connectivity patterns within the graph such as community structure, number of spanning trees, etc. [1, 2]. We used normalized Laplacian as its eigenvalues are bounded in the range $[0, 2]$, and thus makes it a more stable and preferable representation [2]. Also, note that while spectral distance is permutation invariant (i.e. invariant to ordering of nodes), Hamming distance and DeltaCon distance preserve node identities. Consequently, the spectral distance between two isomorphic graphs is zero, and thus not best suited for applications where we want to preserve node (i.e., genomic loci) identities. However, because it captures differences in global connectivity patterns, it can still be useful and we include it in our analysis. The fast belief propagation matrix used in DeltaCon distance is designed to model the diffusion of information throughout a graph [3]. The entry $S_{i,jk}$ of the matrix $\mathbf{S}_i$ indicates the influence that node j has on node k. Intuitively, node j has more influence/affinity to node k if there are many, short heavily-weighted paths from node j to k. Thus, DeltaCon, while preserving node identities, captures differences in global connectivity patterns (in terms of how information flows between any two nodes) in the graphs, and thereby attempts to capture both local and global structural differences.

Supplementary Note 2: Permutation Test for HDM. In order to assess statistical significance while comparing two hypergraphs G_1 and G_2 using hypergraph distance, we pose it as a hypothesis testing problem [4], with:

- Null (H_0): G_1 and G_2 are similar,
- Alternative (H_1): G_1 and G_2 are dissimilar.

Since the null distribution for H_0 is unknown, we use a permutation test to empirically estimate it. Let D be any hypergraph distance, and let $0 < p_s < 1$ be the desired significance level of the test. The steps in the permutation test involve:

- Step 1: Randomly generate a family of hypergraphs $\{\mathsf{R}_i\}_{i=1}^{N}$ which are similar to G_1, and compute $\tilde{D}_i = D(\mathsf{G}_1, \mathsf{R}_i), i = 1, \cdots, N$.
- Step 2: Compute $D_{12} = D(\mathsf{G}_1, \mathsf{G}_2)$.
- Step 3: Compute p-value as $p = \frac{1}{N}\sum_{i=1}^{N} \mathcal{I}_{D_{12}}(\tilde{D}_i)$, where $\mathcal{I}$ is indicator function, with $\mathcal{I}_z(x) = 1$ if $x > z$ and $\mathcal{I}_z(x) = 0$ otherwise.
- Step 4: Reject H_0 if $p \leq p_s$.

In Step 1, we follow a Chung–Lu (CL) procedure described in [5] to generate R_i with similar vertex degree and edge size distribution as of G_1. Let $d_{1v} = (d(v_1), \cdots, d(v_n))$ and $d_{1e} = (d(e_1), \cdots, d(e_{m_1}))$ be vertex degree and edge size distribution vectors of $\mathsf{G}_1 = (\mathbf{V}_1, \mathbf{E}_1)$, where, $d(v_i)$ is degree of node $v_i \in \mathbf{V}_1$ $i = 1, \cdots, n$ and $d(e_j)$ is order of edge $e_j \in \mathbf{E}_1, j = 1, \cdots, m_1$. In order to be realizable by a hypergraph, vertex degree and edge size distribution should satisfy $c = \sum_{i=1}^{n} d(v_i) = \sum_{i=1}^{m_1} d(e_i)$. As per this procedure, the probability that a vertex belongs to a hyperedge in R is proportional to the product of the desired vertex degree and edge size, i.e.,

$$\mathcal{P}(u \in e) = \frac{d(u)d(e)}{c}$$

To ensure this probability is always less than 1, one may further require that the input sequences satisfy $\max_{i,j} d(u_i)d(e_j) \leq c$.

3 Supplementary Figures

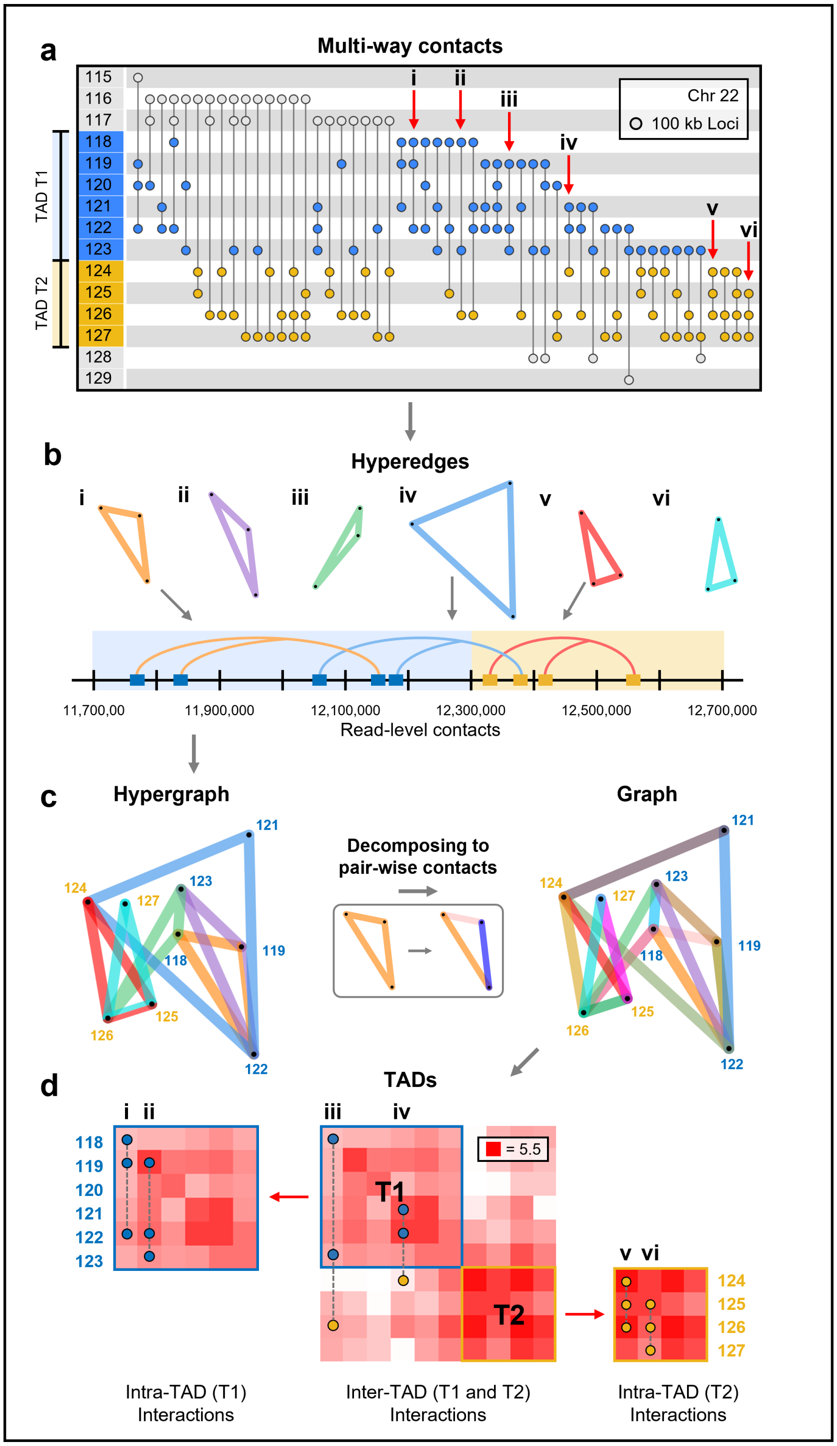

Supplementary Figure 1: Local organization of the genome. **a** Incidence matrix visualization of a region in Chromosome 22 from B lymphocytes. The numbers in the left column represent genomic loci, vertical lines represent multi-way contacts, where nodes indicate the corresponding locus' participation in this contact. The blue and yellow regions represent two TADs, T1 and T2. The six contacts, denoted by the labels i-vi, are used as examples for hypergraph and genomic folding pattern visualizations. **b** Hyperedge and read-level visualizations of the multi-way contacts i-vi from the incidence matrix in a. Blue and yellow shaded areas (bottom) indicate which TAD each locus corresponds to. **c** Hypergraph visualization of the multi-way contacts i-vi from a. Blue and yellow labels indicate which TADs these loci participate in. **d** Contact frequency matrices were constructed by separating all multi-way contacts within and between the two TADs into their pairwise combinations. Example multi-way contacts are superimposed onto contact frequency matrices. All multi-way contacts in this figure were determined in 100 kb resolution after noise reduction (Methods).

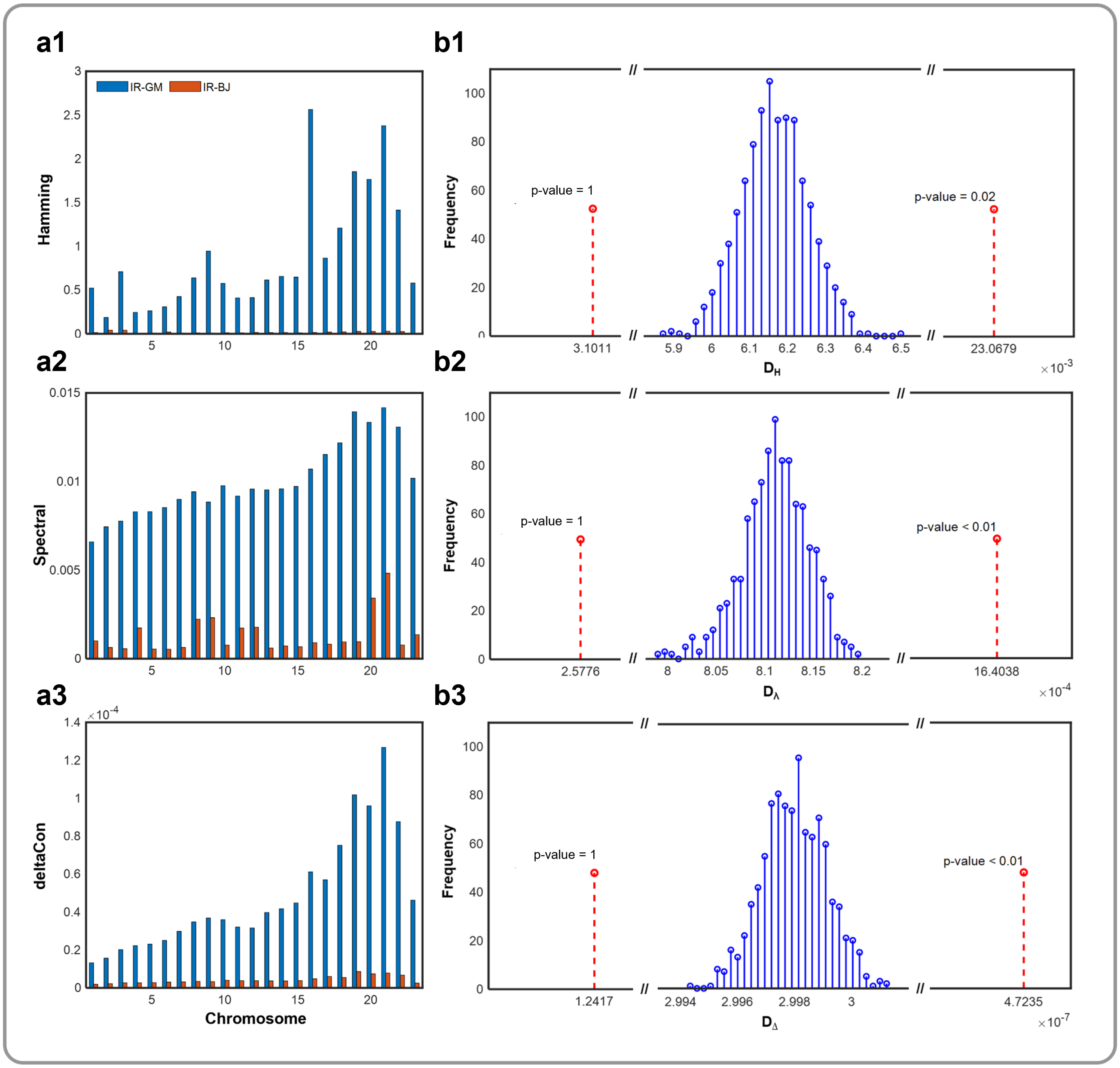

Supplementary Figure 2: Hypergraph distance between genome-wide hypergraphs derived from adult fibroblasts, neonatal fibroblasts, and B lymphocytes. **a1-3** Hypergraph distance measures computed at the chromosome level between adult fibroblasts and B lymphocytes (blue) and between adult and neonatal fibroblasts (orange). **b1-3** Null distribution formed by measuring the hypergraph distances between the hypergraph derived from adult fibroblasts and random hypergraphs. The actual distance between the adult fibroblast and neonatal fibroblast (left red dashed line) as well as adult fibroblast and B lymphocyte (right red dashed line) genome-wide hypergraphs is highlighted with p-values denoted. IR = adult fibroblasts; BJ = neonatal fibroblasts; GM = B lymphocytes. P-values were derived using a one-sided permutation test and are indicated in each panel.

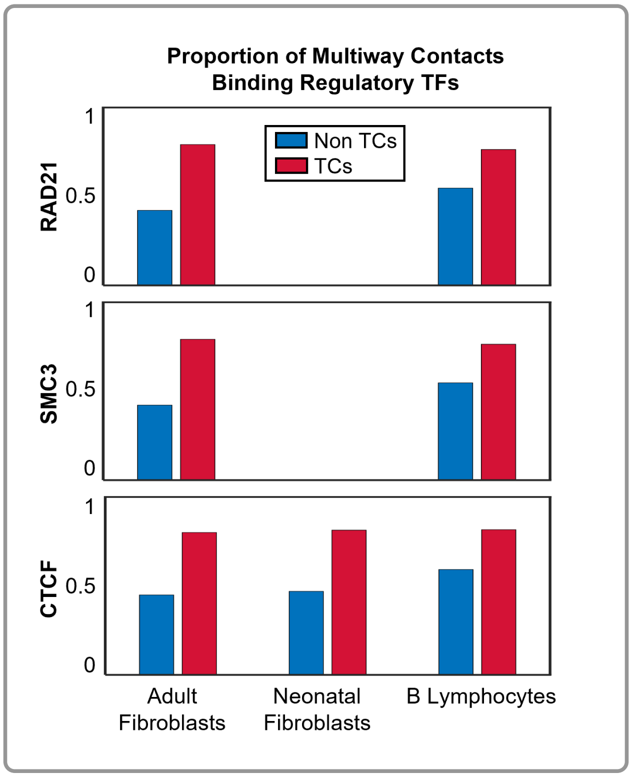

Supplementary Figure 3: Multi-way contacts with regulatory TF binding. CTCF, a common transcription factor responsible for regulating transcription and chromatin folding, and associated cohesin subunits - RAD21 and SMC3 - all exhibited preferential binding to transcription clusters (TCs) across cell types rather than multi-way contacts not classified as transcription clusters (non TCs). RAD21 and SMC3 ChIP-seq data for the BJ cell line (neonatal fibroblasts) were not available.

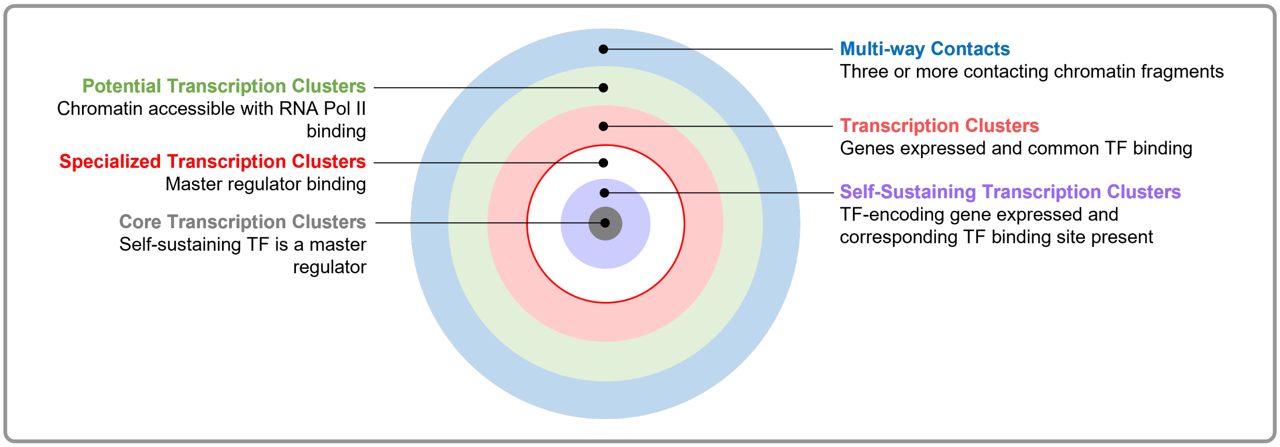

Supplementary Figure 4: Overview of multi-way contact classifications.

4 Supplementary Tables

File Type	Description
.fastq	Text file which contains unique identifiers for Pore-C reads and the raw sequences contained within each read
.pairs	Text file which contains pairs of aligned genomic loci at base-pair resolution, which can be grouped together using unique identifiers to construct multi-way contacts
.parquet	Text file which contains aligned multi-way contacts at base-pair resolution, restriction fragment assignments, and indicators for the quality of read alignment
.mcool	Binary storage file which contains pairwise interactions from Pore-C data at multiple resolutions

Supplementary Table 1: Descriptions of file types used within the computational framework (Figure 1).

Order	Multi-way Contacts	Transcription Clusters	Clusters with $\geq$ 2 Genes	Clusters with $\geq$ 1 Enhancer	Clusters with Common TFs	Clusters with Common MRs
3	240,477	8,384	4,157	3,487	3,179	3,063
	301,366	8,182	7,518	358	336	333
	379,165	11,261	7,518	7,358	6,742	6,598
4	227,352	4,345	2,686	2,387	2,172	2,088
	156,742	2,593	2,008	163	150	148
	181,554	3,254	2,658	2,638	2,495	2,443
5	196,423	1,996	1,434	1,315	1,015	885
	98,172	999	834	358	336	333
	98,272	1,021	877	869	672	587
6+	1,000,231	1,802	1,419	1,343	892	757
	178,705	590	549	68	61	59
	142,575	544	514	513	385	336

Supplementary Table 2: Summary of multi-way contacts with enhancers. Multi-way contacts from B lymphocytes (white rows), neonatal fibroblasts (light gray rows), and adult fibroblasts (dark gray rows, V1-V4) are listed after different filtering criteria. Multi-way contacts are considered to be potential transcription clusters if all loci within the multi-way contact are accessible and at least one locus has binding from RNA Pol II. These multi-way contacts are then queried for nearby expressed genes and enhancers. If a transcription cluster candidate has at least two expressed genes and at least one enhancer locus, we determine whether the majority of these genes have common transcription factors (TFs) through binding motifs. If only two expressed genes are contained within a transcription cluster candidate, we require both genes to have common TFs. From the set of transcription clusters with common TFs, we calculate how many clusters have at least one common master regulator (MR) (Algorithm 6).

**Chr**	**1**	**2**	**3**	**4**	**5**	**6**	**7**	**8**	**9**	**10+**
1	230	8,333	14,492	5,501	2,093	872	353	133	54	57
2	241	9,183	15,548	6,180	2,373	873	437	197	71	95
3	198	7,113	13,631	5,113	1,909	750	302	132	57	50
4	190	6,342	11,147	4,360	1,642	598	284	113	56	28
5	179	5,785	10,059	3,897	1,457	640	249	106	39	31
6	167	5,361	9,368	3,551	1,364	511	207	75	39	27
7	159	4,851	8,365	3,258	1,274	453	180	72	35	26
8	143	4,321	7,596	2,864	1,069	393	154	71	32	16
9	122	2,722	4,899	1,831	614	209	76	31	8	6
10	134	4,010	7,185	2,695	912	337	144	63	23	14
11	133	3,698	6,613	2,533	890	330	101	62	14	14
12	132	3,815	6,630	2,497	878	324	114	47	17	12
13	97	2,627	4,767	1,751	588	247	82	37	11	8
14	87	2,209	3,932	1,366	509	140	51	19	10	2
15	82	1,623	2,812	991	298	98	29	16	2	1
16	80	1,650	3,126	1,181	363	127	39	11	5	2
17	82	1,345	2,374	732	201	66	20	4	3	2
18	77	1,828	3,230	1,128	368	149	51	16	10	4
19	56	873	1,455	462	118	41	7	3	0	0
20	64	1,247	2,238	783	222	69	27	6	3	0
21	39	453	809	264	79	18	6	1	0	0
22	39	438	728	206	43	13	3	0	1	0
X	151	1,078	1,852	882	350	119	43	17	10	0
Y	23	70	93	25	6	4	0	0	0	0

Supplementary Table 3: Adult fibroblast intra-chromosome contact orders (1 Mb resolution).

Chr	1	2	3	4	5	6	7	8	9	10+
1	232	4,725	17,254	16,396	10,752	6,836	4,248	2,663	1,652	3,130
2	243	4,964	16,695	16,702	11,787	7,891	5,172	3,432	2,288	4,631
3	199	4,055	16,351	14,795	9,846	6,376	4,023	2,691	1,759	3,293
4	191	3,725	13,399	13,309	9,192	6,218	4,081	2,632	1,698	3,301
5	180	3,449	12,352	12,458	8,799	5,810	3,672	2,358	1,548	2,728
6	171	3,300	11,249	11,640	7,862	5,307	3,235	2,139	1,376	2,486
7	160	3,086	11,350	11,272	7,758	5,101	3,223	2,213	1,391	2,613
8	145	2,895	10,755	10,608	7,333	4,778	3,141	2,068	1,289	2,477
9	124	2,399	9,360	8,535	5,847	3,802	2,393	1,572	931	1,753
10	134	2,580	9,429	9,273	6,203	4,113	2,576	1,632	1,020	1,856
11	134	2,505	8,979	8,661	5,795	3,629	2,250	1,408	852	1,530
12	134	2,376	8,329	8,200	5,465	3,449	2,192	1,344	833	1,304
13	99	1,763	6,665	6,527	4,585	2,935	1,834	1,078	635	1,159
14	89	1,531	5,909	5,884	3,863	2,236	1,430	858	514	843
15	85	1,349	5,064	5,130	3,354	1,964	1,164	706	372	582
16	83	1,444	5,993	5,722	3,769	2,437	1,419	808	494	813
17	84	1,355	4,278	4,294	2,612	1,514	882	475	271	366
18	80	1,478	6,015	5,569	3,616	2,359	1,380	861	549	840
19	57	884	3,335	3,251	1,998	1,141	664	383	224	252
20	65	1,053	4,344	4,149	2,684	1,656	902	547	315	441
21	40	481	2,285	2,245	1,392	747	402	235	96	127
22	39	445	1,739	1,795	1,098	512	255	127	59	72
X	156	3,681	12,552	11,686	8,206	5,915	3,941	2,738	1,950	4,414

Supplementary Table 4: GM12878 intra-chromosome contact orders (1 Mb resolution).

**Chr**	**1**	**2**	**3**	**4**	**5**	**6**	**7**	**8**	**9**	**10+**
1	230	8,112	12,567	5,543	2,385	1,008	442	184	81	52
2	241	9,312	14,641	6,579	3,000	1,316	594	266	106	73
3	197	7,494	11,813	5,199	2,333	978	423	167	76	53
4	190	6,946	10,900	4,973	2,205	924	363	131	60	48
5	179	6,084	9,605	4,397	1,904	772	315	116	57	29
6	166	5,676	9,083	3,992	1,648	647	270	108	41	29
7	159	5,044	8,005	3,582	1526	619	265	101	40	27
8	143	4,538	7,273	3,218	1,339	499	219	82	38	21
9	123	2,746	4,622	2,048	742	275	98	48	7	4
10	134	3,989	6,336	2,894	1,148	507	144	64	29	10
11	134	3,846	6,275	2,758	1,045	414	169	71	18	22
12	133	3,825	6,138	2,622	1,063	422	153	54	15	12
13	97	2,861	4,844	2,097	803	322	114	41	14	1
14	87	2,308	3,816	1,609	595	217	77	26	11	6
15	80	1,566	2,575	980	334	99	34	7	4	0
16	80	1,523	2,859	1,221	444	118	48	16	8	2
17	80	1,237	2,111	767	227	76	28	5	1	0
18	77	1,905	3,332	1,433	509	147	56	19	2	2
19	56	783	1,197	407	122	37	9	3	1	0
20	64	1,277	2,179	884	319	96	34	10	0	2
21	39	482	887	347	100	30	6	1	1	0
22	39	436	706	219	49	8	5	1	0	0
X	151	1,475	2,486	1,231	454	203	80	25	9	6
Y	23	89	137	37	9	2	0	0	0	0

Supplementary Table 5: Neonatal fibroblast intra-chromosome contact orders (1 Mb resolution).

Neonatal Fibroblast		Adult Fibroblast		B Lymphocyte	
**ZNF2**	37.2%	**ZNF2**	36.2%	**HINFP**	34.1%
**SP2**	36.6%	**ZNF441**	35.6%	**ZNF441**	28.7%
**HINFP**	35.5%	**HINFP**	34.4%	**DNMT1**	28.4%
**ZNF441**	34.7%	**MBD2**	33.8%	**MBD2**	26.7%
**MBD2**	34.1%	**ZNF486**	32.3%	**KLF7**	26.3%
**TCFL5**	33.0%	**TCFL5**	32.1%	**ZNF141**	25.9%
**ZNF486**	31.6%	**DNMT1**	31.4%	**EGR2**	25.4%
**DNMT1**	30.9%	**HES1**	30.2%	**ZNF200**	23.8%
**HES1**	30.6%	**HES4**	30.2%	**CXXC1**	23.6%
**HES4**	30.6%	**EGR1**	30.2%	**ZNF383**	22.4%
**ZNF202**	29.3%	**ZNF202**	29.0%	**ZBTB14**	22.3%
**EGR1**	29.3%	**ZBTB14**	28.5%	**TCFL5**	21.9%
**ZBTB14**	28.6%	**ZNF200**	28.3%	**ZNF100**	21.7%
**KLF7**	27.9%	**EGR2**	28.0%	**ZNF202**	21.5%
**ZNF200**	27.8%	**KLF7**	28.0%	**E2F6**	20.6%

Supplementary Table 6: Top 15 most frequently observed TF binding sites across transcription clusters.

	Transcription Cluster (Chromosomes)	Gene/TF Analog	Master Regulator?
**Adult Fibroblasts**	[9 19 3 6]	FOXO3	Y
	[19 19 19]	ZNF134	Y
	[1 1 1]	PBX1	Y
	[18 20 18]	MBD2	Y
	[19 10 19]	[MZF1,ZNF33A]	Y
	[6 2 6]	ATOH8	Y
	[19 18 19 19 18]	TCF4	N
	[19 19 19]	ZNF320	N
	[15 15 15 15 12]	ZNF740	N
**Neonatal Fibroblasts**	[19 17 19]	ZNF582	Y
	[17 17 17]	STAT3	Y
	[11 11 21]	ETS1	Y
	[6 3 17 1 8]	STAT3	Y
	[19 19 19 19 19]	TCF3	N
	[9 16 13]	ZNF821	N
	[16 7 11 7 17]	ZNF263	N
	[19 1 4 14]	NFIA	N
**B Lymphocytes**	[16 16 16 12]	MAZ	Y
	[7 16 1 6]	TFAP4	Y
	[19 19 5 19]	ZNF296	Y
	[19 19 19]	ZNF543	Y
	[7 7 7 7]	ZNF783	Y
	[19 19 19 19]	ZBTB7A	Y
	[6 6 4]	HIVEP1	Y
	[19 19 19 19]	ZNF574	Y
	[6 6 6]	ZBTB12	Y
	[17 17 19]	STAT3	Y
	[10 8 8 12]	EGR2	N
	[15 19 19 19 19]	ZNF555	N
	[19 19 15 19 19 19]	ZNF555	N

Supplementary Table 7: Self-Sustaining Transcription Clusters. All transcription clusters where a TF (specifically its binding motif) and its corresponding TF-encoding gene are both found. The 'Transcription Cluster' column contains the multi-way contacts within and/or between chromosomes that make up the transcription clusters. The 'Gene/TF' column contains the shared TF and TF-encoding gene name. The 'Master Regulator' column denotes whether the TF in the previous column is a master regulator.

Chromosome	IR	BJ	GM
1	5.4289	5.4327	5.4337
2	5.4800	5.4799	5.4808
3	5.2788	5.2783	5.2809
4	5.2308	5.2409	5.2386
5	5.1760	5.1756	5.1784
6	5.1065	5.1064	5.1272
7	5.0568	5.0566	5.0607
8	4.9497	4.9639	4.9689
9	4.7871	4.8037	4.8118
10	4.8846	4.8842	4.8823
11	4.8767	4.8837	4.8964
12	4.8769	4.8839	4.8808
13	4.5591	4.5589	4.5778
14	4.4490	4.4486	4.4698
15	4.3736	4.3729	4.4219
16	4.3747	4.3734	4.4116
17	4.3847	4.3832	4.4085
18	4.3245	4.3246	4.3757
19	4.0167	4.0154	4.0182
20	4.1367	4.1521	4.1517
21	3.6277	3.6548	3.6573
22	3.6268	3.6272	3.6307
X	4.9887	4.9923	5.0380
**Whole Genome**	**7.9744**	**7.9762**	**7.9784**

Supplementary Table 8: Hypergraph Entropy. Chromosome-level genomic hypergraphs for adult fibroblasts (IR), neonatal fibroblasts (BJ) and B lymphocytes (GM). Last row is the genome-wide hypergraph entropy for IR, BJ, and GM.

References

[1] Fan R. K. Chung and Fan Chung Graham. Spectral graph theory. Number 92. American Mathematical Soc., 1997.

[2] Claire Donnat and Susan Holmes. Tracking network dynamics: A survey using graph distances. The Annals of Applied Statistics, 12(2):971 – 1012, 2018.

[3] Danai Koutra, Joshua T Vogelstein, and Christos Faloutsos. Deltacon: A principled massive-graph similarity function. In Proceedings of the 2013 SIAM international conference on data mining, pages 162–170. SIAM, 2013.

[4] Amit Surana, Can Chen, and Indika Rajapakse. Hypergraph dissimilarity measures. arXiv preprint arXiv:2106.08206, 2021.

[5] Sinan G Aksoy, Cliff Joslyn, Carlos Ortiz Marrero, Brenda Praggastis, and Emilie Purvine. Hypernetwork science via high-order hypergraph walks. EPJ Data Science, 9(1):16, 2020.
