# Supplementary material for: Deciphering multi-way interactions in the human genome
Source: Nat Commun. 2022 Sep 20;13:5498. doi: 10.1038/s41467-022-32980-z (PMC9489732; doi:10.1038/s41467-022-32980-z)
Supplement: Supplementary file 2 — Reporting Summary [file 41467_2022_32980_MOESM2_ESM.pdf]

nature portfolio

Corresponding author(s): Indika Rajapakse

Last updated by author(s): Aug 10, 2022

Reporting Summary

Nature Portfolio wishes to improve the reproducibility of the work that we publish. This form provides structure for consistency and transparency in reporting. For further information on Nature Portfolio policies, see our Editorial Policies and the Editorial Policy Checklist.

Statistics

For all statistical analyses, confirm that the following items are present in the figure legend, table legend, main text, or Methods section.

n/a	Confirmed	
☐	☒	The exact sample size (*n*) for each experimental group/condition, given as a discrete number and unit of measurement
☐	☒	A statement on whether measurements were taken from distinct samples or whether the same sample was measured repeatedly
☐	☒	The statistical test(s) used AND whether they are one- or two-sided<br>*Only common tests should be described solely by name; describe more complex techniques in the Methods section.*
☒	☐	A description of all covariates tested
☒	☐	A description of any assumptions or corrections, such as tests of normality and adjustment for multiple comparisons
☐	☒	A full description of the statistical parameters including central tendency (e.g. means) or other basic estimates (e.g. regression coefficient) AND variation (e.g. standard deviation) or associated estimates of uncertainty (e.g. confidence intervals)
☐	☒	For null hypothesis testing, the test statistic (e.g. *F*, *t*, *r*) with confidence intervals, effect sizes, degrees of freedom and *P* value noted<br>*Give P values as exact values whenever suitable.*
☒	☐	For Bayesian analysis, information on the choice of priors and Markov chain Monte Carlo settings
☒	☐	For hierarchical and complex designs, identification of the appropriate level for tests and full reporting of outcomes
☒	☐	Estimates of effect sizes (e.g. Cohen's *d*, Pearson's *r*), indicating how they were calculated

Our web collection on statistics for biologists contains articles on many of the points above.

Software and code

Policy information about availability of computer code

Data collection	Base calling was performed on the Oxford Nanopore GridiON using Guppy (version 4.0.11). The Pore-C Snakemake pipeline (commit 6b2f762) was used for genome alignment and mapping.
Data analysis	The source code of packages used in these analyses can be accessed at following links along with dependencies. MATLAB R2021b was used for developing these tools<br>http://github.com/lindsly/Pore-C_Hypergraphs<br>https://github.com/nanoporetech/Pore-C-Snakemake<br>https://aviz.fr/paohvis

For manuscripts utilizing custom algorithms or software that are central to the research but not yet described in published literature, software must be made available to editors and reviewers. We strongly encourage code deposition in a community repository (e.g. GitHub). See the Nature Portfolio guidelines for submitting code & software for further information.

Data

Policy information about availability of data

All manuscripts must include a data availability statement. This statement should provide the following information, where applicable:

- Accession codes, unique identifiers, or web links for publicly available datasets
- A description of any restrictions on data availability
- For clinical datasets or third party data, please ensure that the statement adheres to our policy

All data created in this work is available through computer code's GitHub page (http://github.com/lindsly/Pore-C_Hypergraphs). Publicly available data accession codes are as follows: ENCFF310UDS, ENCFF310UDS, ENCFF410XEP, ENCFF676DGR, ENCFF912DZY, ENCFF203SRF, ENCFF518RUC, ENCFF951PEM, ENCSROOOEFJ, ENCSROOOEAC, ENCSROOOHPG, ENCSROOODZP, ENCFF353SBP, ENCFF496RIW, ENCFF477JDG, ENCFFOOSWBQ ENCFF306TLL, ENCFF418FIT. Web links to publicly available data are as follows: http://www.enhanceratlas.org/, https://github.com/nanoporetech/pore-c/.

Human research participants

Policy information about studies involving human research participants and Sex and Gender in Research.

Reporting on sex and gender	One male donor provided adult primary fibroblasts via skin biopsy
Population characteristics	N/A
Recruitment	N/A
Ethics oversight	The IR skin collection was collected under the University of Michigan Human Subjects Study HUM00135011 "Fibroblast Harvesting and Culture"

Note that full information on the approval of the study protocol must also be provided in the manuscript.

Field-specific reporting

Please select the one below that is the best fit for your research. If you are not sure, read the appropriate sections before making your selection.

☒ Life sciences ☐ Behavioural & social sciences ☐ Ecological, evolutionary & environmental sciences

For a reference copy of the document with all sections, see nature.com/documents/nr-reporting-summary-flat.pdf

Life sciences study design

All studies must disclose on these points even when the disclosure is negative.

Sample size	2.5 million cells were processed as one sample for each cell type
Data exclusions	No sample was excluded
Replication	We performed four sequencing runs for IR fibroblasts and BJ fibroblasts
Randomization	N/A
Blinding	N/A

Reporting for specific materials, systems and methods

We require information from authors about some types of materials, experimental systems and methods used in many studies. Here, indicate whether each material, system or method listed is relevant to your study. If you are not sure if a list item applies to your research, read the appropriate section before selecting a response.

Materials & experimental systems

n/a	Involved in the study
x	Antibodies
	x Eukaryotic cell lines
x	Palaeontology and archaeology
x	Animals and other organisms
x	Clinical data
x	Dual use research of concern

Methods

n/a	Involved in the study
x	ChIP-seq
x	Flow cytometry
x	MRI-based neuroimaging

Eukaryotic cell lines

Policy information about cell lines and Sex and Gender in Research

Cell line source(s)	IR: adult primary fibroblasts via skin biopsy<br>BJ: human foreskin fibroblasts (Cat no. CRL-2522, ATCC)
Authentication	Fibroblast cells were harvested by skin punch biopsy and cell type was confirmed by morphology
Mycoplasma contamination	Cell lines were not tested for mycoplasma contamination
Commonly misidentified lines (See ICLAC register)	No commonly misidentified lines were used in this study
